# Case report: Turcot syndrome type 2 in a developing country within the Caribbean

**DOI:** 10.3389/fonc.2024.1331271

**Published:** 2024-03-14

**Authors:** Melissa Daniel-Abdool, Brandon Griffith, Ute Bartels, Curt Bodkyn, Kevon Dindial

**Affiliations:** ^1^ Faculty of Medical Sciences, The University of the West Indies, St. Augustine, Trinidad and Tobago; ^2^ Division of Haematology/Oncology, Hospital for Sick Children (SickKids), Toronto, ON, Canada; ^3^ Department of Clinical Medical Sciences, The University of the West Indies, St. Augustine, Trinidad and Tobago; ^4^ Department of Pediatrics, Eric Williams Medical Sciences Complex, San Juan, Trinidad and Tobago

**Keywords:** medulloblastoma, Turcot syndrome, cancer predisposition syndromes, colorectal carcinoma, APC, low-and-middle-income countries, Caribbean, pediatrics

## Abstract

Medulloblastoma is the most common malignant pediatric brain tumor and has been linked to known cancer predisposition syndromes. We report a case of medulloblastoma of a 12-year-old Indo-Trinidadian female with a strong family history of colorectal carcinoma. In collaboration with the SickKids-Caribbean Initiative (SCI), her tumor was confirmed to be a WHO grade 4 medulloblastoma – Wnt subtype. Genetic testing further confirmed the presence of a pathogenic APC gene variant [c.3183_3187del (p.Gln1062*)] which led to a diagnosis of Turcot syndrome type 2. The index patient received multimodal therapy which included surgery, radiation and chemotherapy and is currently post end-of-treatment and in remission. This case report aims to highlight the complexity of diseases and the need for expertise in identifying them in low-and-middle income countries, the need for access to specialized testing and the benefits of collaborating between low-and-middle income and high-income countries when managing complex oncology patients.

## 1 Introduction

Cancer is a major leading cause of disease-related deaths for children worldwide, with an increasing trend in recent decades. Despite this prevalence, the five-year survival rate of these patients exceeds 80% – largely due to improvements in diagnostic imaging, technology and multi-drug regimens (1). However, outcomes vary between high-income countries (HICs) and low-middle income countries (LMICs) due to multiple factors which include constraints to equitable health due to inadequate funding; shortages in appropriately trained healthcare professionals; limited and/or unreliable access to specialized testing and essential medicines; and lack of registries and case tracking to facilitate appropriate resource planning and policy generation (2). The most common type of pediatric solid tumors are brain tumors classified as supratentorial or infratentorial in location. Medulloblastomas are the most common malignant pediatric brain tumors and are defined to occur infratentorial within the posterior fossa (3).

The etiology of childhood cancer is multifactorial with both intrinsic and extrinsic factors contributing to its development. Intrinsic factors involve a genetic predisposition that increases the risk of specific diseases regardless of environmental factors.

To date, a variety of genetic predisposition syndromes are associated with primary brain tumors. Common syndromes are Neurofibromatosis type 1 and 2, Tuberous sclerosis, Li Fraumeni syndrome, Gorlin’s syndrome (nevoid basal cell carcinoma syndrome), familial adenomatous polyposis and its associations with Turcot syndrome (TS) and Gardner syndrome, rhabdoid tumor predisposition syndrome and retinoblastoma germline syndrome or hereditary retinoblastoma (1, 4).

In this article, we focus on familial adenomatous polyposis (FAP) and TS. TS was originally described by Canadian surgeon Jacques Turcot in 1959. Turcot reported on two consanguineous siblings with a combination of colon polyposis and primary brain tumor. Anecdotally, the brother presented with a case of polyposis, sigmoid colon adenocarcinoma and medulloblastoma and the sister presented with glioblastoma and pituitary adenoma (5). TS can be further classified as TS type 1 (TS1) (primary central nervous system tumor secondary to mismatch repair [MMR] gene variant) or TS type 2 (TS2) (primary central nervous system tumor secondary to adenomatous polyposis coli [APC] gene variant). Colorectal carcinoma (CRC) is often associated with TS and is divided by hereditary non-polyposis colorectal cancer (HNPCC) and familial adenomatous polyposis (FAP), each associated with MMR and APC gene variants respectively.

We aim to highlight the role of international collaborations between HICs and LMICs in identifying and managing complex diagnoses which ultimately leads to more superior outcomes. In our index case, we specifically highlight the collaboration between The Hospital for Sick Children (SickKids) in Canada and Eric Williams Medical Sciences Complex, Trinidad and Tobago, made possible through the SickKids-Caribbean Initiative (SCI). The aim of SCI is to improve the outcomes and quality of life for children with cancer and blood disorders throughout six partnering Caribbean countries — The Bahamas, Barbados, Jamaica, St. Lucia, St. Vincent and the Grenadines and Trinidad and Tobago — that may not have the resources to maximize care (2).

## 2 Case history

The index case is a 12-year-old Indo-Trinidadian female who presented to the emergency department with a four-day history of intermittent vomiting and one instance of decreased consciousness. She was noted to have a five-month history of headaches and myopia prior to presentation, and a three-week history of persistent fatigue and progressive gait unsteadiness. One week prior to presentation, the patient complained of diplopia, bilateral tinnitus, throbbing lateral and frontal headaches, had decreased appetite and periodic vomiting. She had a history of congenital right-sided hemi-hypertrophy and Melker-Rossenthal syndrome that was monitored with routine follow-up care, but otherwise exhibited normal physical and psychosocial development throughout childhood.

**Figure 1:**
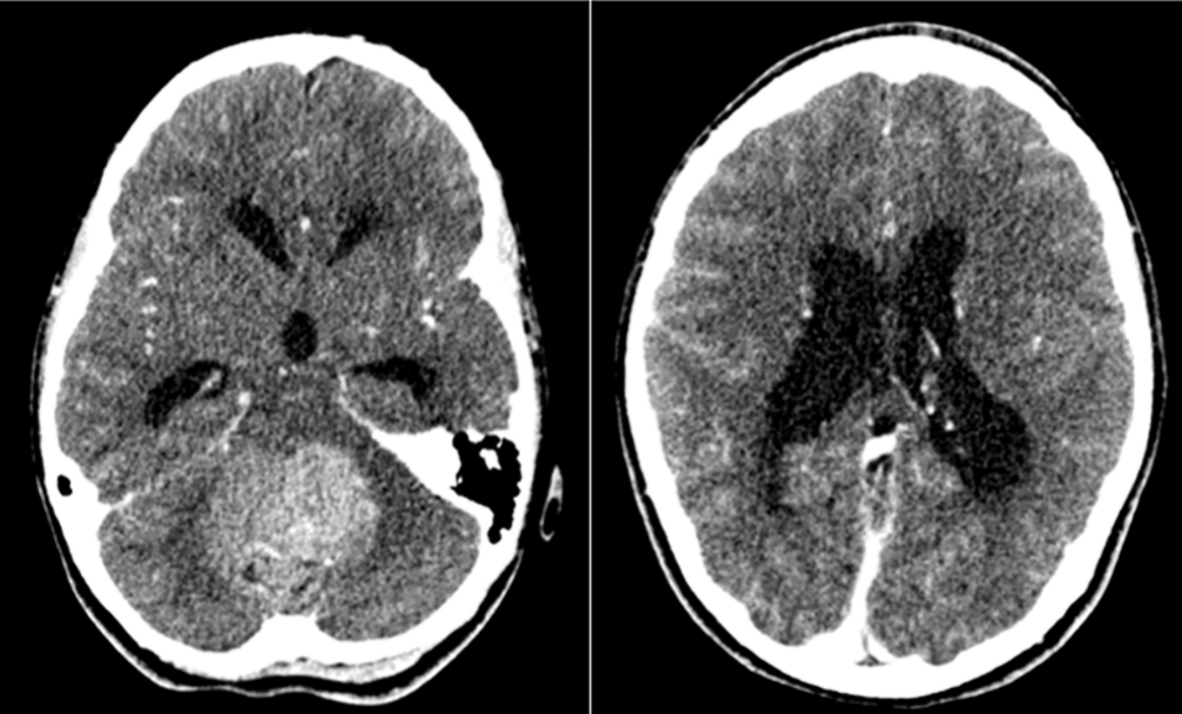
Axial post-contrast CT images demonstrating a large heterogenous mass arising from the posterior fossa (left image) and obstructive hydrocephalus secondary to the mass (right image) in our index case at time of initial presentation.

**Figure 2:**
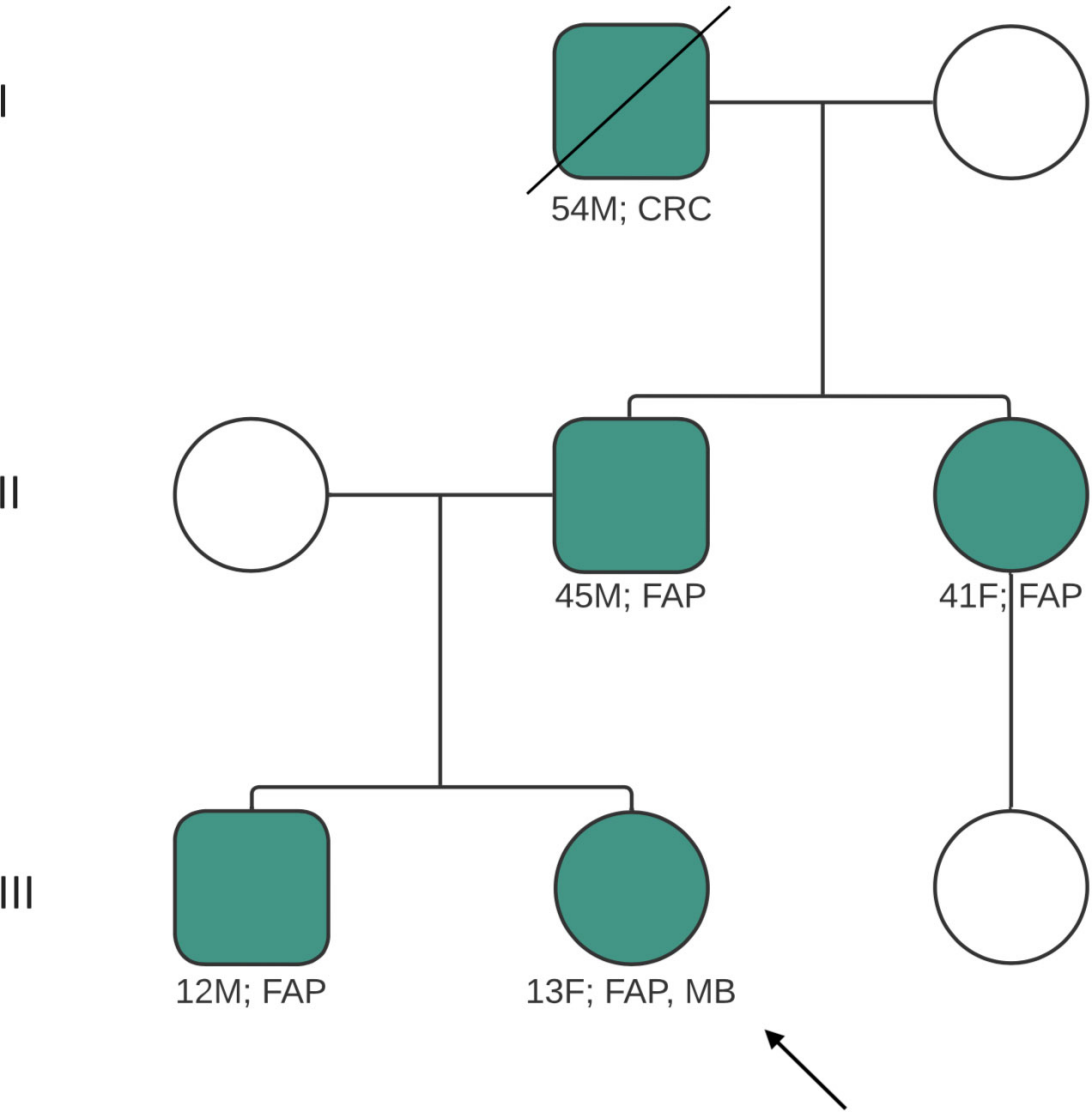
Pedigree of patient’s family, illustrating family members affected and/or diagnosed with FAP, CRC. Index case indicated by arrow. Diagonal line indicates deceased relative. CRC, colorectal carcinoma; FAP, familial adenomatous polyposis; MB, medulloblastoma; M, male; F, female.

Her initial assessments included neuroimaging scans which showed a cerebellar mass with obstructive hydrocephalus and diffuse effacement of the sulci, resulting in raised intracranial pressure (ICP) (**Figure 1**). A ventriculoperitoneal shunt was sited and she was referred to the Eric Williams Medical Sciences Complex where the primary neurosurgeon and pediatric hematologist/oncologist were based. On initial examination, the patient had an inability to ambulate without support, combined with left-sided weakness, a right convergent squint and aphasia – all in keeping with posterior fossa syndrome. On initial consultation with the pediatric oncologist, a family history of CRC and clinical FAP (**Figure 2**) was elicited. Her paternal grandfather was diagnosed with CRC at 53-years-old and this prompted subsequent investigations into the patient’s father and paternal aunt via colonoscopy. They were both identified as having multiple colonic polyps and thus, both were clinically diagnosed with FAP. They each underwent prophylactic total colectomies at ages 37 and 32, respectively, in an effort to reduce the risk of developing CRC in the future. There are two other paternal 2^nd^ degree relatives with a history of malignancy, including CRC while two maternal 2^nd^ degree relatives have a history of malignancy including breast and lung cancer. Both of the patient’s paternal and maternal great-grandfathers developed prostate cancer, indicating a strong, multi-generational family history of cancer.

To identify a potential genetic link, the patient underwent focused genetic testing locally and a pathogenic variant in the APC gene was detected, confirmed as variant c.3183_3187del (p.Gln1062*). The patient and her family were counseled on the mode of inheritance of this variant and its likely link in developing medulloblastoma. Subsequent cascade testing was done for her first-degree relatives which included her father, brother and paternal aunt. All three relatives were found to have identical pathogenic variation in the APC gene, confirming the clinical suspicion of the genetic cancer predisposition of FAP.

Meanwhile, the patient underwent neurosurgery for attempted gross total resection shortly after her initial pediatric oncology consultation. A subtotal resection was achieved given the complexity of the tumor and high risk of post-surgical morbidities. The resected tumor was sent to both our local pathology laboratory and SickKids in Canada, facilitated through the SCI. Pathology report at both centers confirmed a WHO grade 4 medulloblastoma while molecular categorization conducted at SickKids identified Wnt-activated subgroup. Microscopic findings showed a cellular neoplasm with mid-size hyperchromatic, round, clear nuclei, fine chromatin and some distinct nucleoli. A subtle nodular pattern was noted within the neoplasm, in addition to Homer-Wright rosettes. The patient had staging workup completed including postoperative MRI brain, spine and cerebrospinal fluid analysis which was negative for malignant cells and hence stage M0. However, due to a residual tumor volume being greater than 1.5cm^2^, the patient was deemed high risk.

Subsequently, our index patient was managed with cranio-spinal radiation (CSI) with a posterior fossa boost as part of standard medulloblastoma treatment with weekly intravenous vincristine chemotherapy. Due to incomplete resections and associated high risk, she received 30 fractions of radiation therapy over the course of six weeks (36Gy in 20 fractions followed by a posterior fossa boost 18Gy in 10 fractions to a total of 54Gy in 30 fractions over a six-week period). After which, the patient started chemotherapy following the Children’s Cancer Intergroup Study with Pediatric Oncology Group, Regimen B Maintenance Chemotherapy, A9961. This protocol consists of eight 42-day cycles consisting of vincristine, cyclophosphamide, cisplatin and G-CSF. To date, the patient has regained the ability to walk short distances with assistance, fine motor skills (legible penmanship and utilizing an electronic tablet), moderate speech and communication abilities and is able to feed herself. She has maintained a positive disposition and plans to continue her secondary education. Her end-of-treatment MRI scans show no active tumor and continues with her multidisciplinary post-treatment management which includes speech therapy, physiotherapy and both she and her brother have been referred to the paediatric gastroenterologist for colonoscopy follow-up. As part of post end-of-treatment follow-up, this patient will be closely monitored for late effects to the multimodal therapy that she received. This includes complete annual endocrine screening and neuropsychological assessment to evaluate her ability to integrate back into school and determine if special aids are needed. As expected, extensive exposure to cisplatin has affected her hearing and caused high-frequency sensorineural hearing loss, which was identified by recent audiometry screening. She has been referred to the otolaryngologist for monitoring and management, in addition to the aforementioned therapies.

## 3 Discussion

Central nervous system tumors comprise approximately 23% of primary pediatric malignancies (1) (**Table 1**). Moreover, tumors can be caused by various factors such as genetic variants or infections. For example, gliomas are categorized by low-grade or high-grade gliomas. Individuals with tuberous sclerosis or neurofibromatosis type 1 have predisposition to developing low-grade gliomas, in addition to complex germline variants in the *BRAF* gene or mitogen-activated protein kinase (MAPK) signaling pathway (such as *NF1* and *RAF* varaints) (4, 10). High-grade gliomas involve abnormalities in multiple pathways such as the phosphatidyl-inositol 3-kinase (PI3K), p53 and retinoblastoma tumor suppressor pathways, and variants of the receptor tyrosine kinase (*RTK)* gene (4, 11). Diffuse intrinsic pontine gliomas involve variants in histone genes such as H3K27M, H3F3A or HIST1H3B (1). Ependymomas may arise from infection with simian vacuolating virus 40 (SV40), neurofibromatosis type 2, Li-Fraumeni syndrome or Turcot syndrome type B/Turcot syndrome 2 (1, 10–12).

**Table 1:** Cancer predisposition genes and associated brain tumor characteristics.

Syndrome	Gene abnormality	Characteristics of CNS lesions	Additional findings	References
Neurofibromatosis Type 1	*NF1*	Low-grade gliomas (optic pathway and brainstem)	Café-au-lait spots, lisch nodules, axillary/inguinal freckling	(4, 6)
Neurofibromatosis Type 2	*NF2*	Bilateral acoustic schwannomas, meningiomas, ependymomas	Increased risk of cataracts and seizures	(2)
Tuberous Sclerosis	*TSC1*; *TSC2*	Subependymal giant cell astrocytoma (SEGA)	Increased risk of skin and renal growths	(2, 3)
Li Fraumeni Syndrome	*TP53*	Malignant glioma, choroid plexus carcinoma	Numerous cancers at younger ages (breast, sarcoma, adrenal cortical carcinoma)	(7)
Gorlin’s Syndrome (nevoid basal cell carcinoma syndrome)	*PTCH*	Medulloblastoma	Basal cell carcinoma	(4, 8)
Familial Adenomatous Polyposis (Gardner/Turcot)	*APC*	Medulloblastoma and malignant glioma	Multiple colonic polyps and increased risk of colorectal carcinoma	(4)
Rhabdoid Tumor Predisposition Syndrome	*SMARCB1*; *SMARCB4* (*INI-1*)	Atypical teratoid rhabdoid tumor (AT/RT)	Rhabdoid tumors in kidney, schwannomatosis; typically < one years of age at diagnosis	(4, 9)
Retinoblastoma (germline)	*RB1*	Trilateral retinoblastoma (unilateral or bilateral retinoblastoma + pineoblastoma)	Pineoblastoma is usually diagnosed after retinoblastoma but often prior to five years of age	(3, 4)

Turcot syndrome (TS) is a rare inherited disorder of colorectal cancer (CRC) with primary brain tumors, as defined by Canadian surgeon Jacques Turcot (13). It can be further classified as TS1 (primary CNS tumor secondary to MMR gene variants) or TS2 (primary CNS tumor secondary to APC gene variants). HNPCC and FAP, each associated with MMR and APC gene variants respectively, are associated with TS (13). Further associations include HNPCC with Lynch syndrome and FAP with Gardner syndrome. With these syndromes and malignancies, the relationship between cancer and genetics is significant such that patients with TS2 and FAP have a 92% relative risk of developing medulloblastoma. TS1 patients tend to be associated with glioblastomas. Additionally, patients with FAP have a nearly 100% relative risk of developing CRC. Nevertheless, TS remains an incredibly rare but life-altering condition. Approximately 30% of CRC patients have a family history of CRC and of these cases, only 3-5% include a genetic component (11, 13–18). To date, there are over 150 cases of TS accounted for in literature. Recordings of the actual number of the cases have been limited by prevalence, the need for genetic testing and the use of various alternative terms such as the more recently suggested substitution of TS with brain tumor polyposis syndrome (BTPS) (13, 17, 19).

Medulloblastomas are the most common brain malignancy of childhood, accounting for 15-20% of all neoplasms of the CNS (20). Histologically, four variants of medulloblastoma can be classified: classic (68-80%); large cell/anaplastic (10-22%), desmoplastic (7%) and extensive nodularity (3%) (12, 21, 22). Our patient has a histological type of vague nodular pattern.

There are also four molecular sub-groups of medulloblastoma which has been classified as: MB-Wnt, MB-Shh (Sonic Hedgehog [Shh]) and Group 3 and 4, of which our patient had MB-Wnt subgroup (12, 19). Turcot syndrome type 2 is associated more commonly with MB-Wnt subtype. This accounts for approximately 10% of diagnoses and is found mainly in girls with peak incidence between 10-12 years of age, as seen in our patient. MB-Wnt has a low tendency to metastasize and patients under 16 years of age have an excellent prognosis due to its prognostic status in comparison to other subgroups (8, 17, 19, 21, 23).

Medulloblastoma are mostly sporadic in nature, however there is an inherited predisposition in TS2 (17, 21, 23). In TS2, the primary brain tumor of medulloblastoma is associated with a germ line variant in the APC gene which is a tumor suppressor gene associated with CRC due to FAP (17, 21). There can be inactivation of the second copy of the gene when there is a germline variant of APC, which appears to be a brain tumorigenesis factor. However, the variant is not always identified in the second copy of the APC gene in brain tumors, and this may be attributed to the difficulty in detecting alterations of the APC gene itself (19). This serves as a point of interest for future research in an effort to increase genetic diagnostic sensitivity in patients with medulloblastoma.

In terms of management, the most illuminating and time-efficient investigation for a primary care provider is the patient’s family medical history. Patients often present with adenomatous polyps at an early, atypical age and most importantly, there is a strong family history of early-onset CRC. In patients with primary CNS tumors and an absence of polyps, any family history of CRC should still raise prompt investigation into genetic syndromes. An understanding that TS1 has autosomal recessive inheritance and TS2 has autosomal dominant inheritance can also be helpful in identifying affected or at-risk individuals in a family tree, as in our case. In our index case, the family history highlighted the autosomal dominance pattern and strong inclination of FAP. By initiating molecular testing and genetic counselling, early diagnosis and preventive management within family members can be maximized.

Due to collaboration with SickKids, facilitated through SCI, our patient had the ability to have a more detailed pathological review of her biopsy tissue, including molecular studies (2). This allowed for her identification of Wnt subtype which is associated with almost all cases of TS2. In addition, multidisciplinary review with the neuro-oncology team was done which helped in guidance of therapeutic approaches for our patient such as considering de-escalation of radiation doses and long-term surveillance screening. As such, this case also highlights the benefits of LMICs collaborating with HICs to facilitate management of complex patients, aid in improving local systems to become more sustainable and eventually enhance the standard of care, particularly highlighting the positive impact that SCI has had on the pediatric oncology landscape within the Caribbean.

### 3.1 Navigating management and surveillance

Genetic counseling in TS is imperative in the management of the patient and their family. Families with confirmed FAP can benefit extensively from counseling due to the risk of developing various tumors. There are no established guidelines for screening, diagnosis and genetic counseling of pediatric patients for rare cancer predisposition syndromes, such as TS (12, 13). When evaluating due to suspicion, gaining details of family history as well as maintaining high vigilance for clinical signs is of utmost importance. In any case, pediatric patients who have 1^st^ degree relatives diagnosed at an early age with CRC with or without associated cancer predisposition syndromes should be screened for pre-cancerous polyps and may require genetic testing. In this case, a pre-adolescent female who developed medulloblastoma and had a family history of CRC prompted genetic testing. This allowed for subsequent identification of identical gene variants in multiple relatives and will provide the basis for surveillance for the patient and her sibling beginning in pre-adolescence.

Confirmation of a cancer predisposition syndrome by molecular diagnosis can impact how the patient and relatives aremanaged, alter when cancer screening may be initiated to detect the syndrome and its manifestations and prevent possible complications by implementing treatment in earlier stages. There has been expansion of pediatric genetic research by the use of molecular testing and analyzation of germlines, as a result of the practice of next generation sequencing (NGS) in evaluating cancer predisposition syndromes in adults.

The panel of multi-gene testing allows the advantage of testing high, moderate and even low penetrance genes associated with the patient’s diagnosis and possible genes of unknown risks. This added benefit may allow for the identification of pathogenic variants of germline variants that are not typically tested for. In this case, our patient had a SMARCE1 mutation which is a variant of uncertain significance, unknown in its associations with TS and whether it may have added to her pathogenicity of disease.

It remains that the mainstay of early evaluation of family members with evidence of FAP, HNPCC-associated APC or MMR gene variants should be early serial colonoscopies and family members of the patient would benefit from genetic analysis. An annual sigmoidoscopy with the goal of identifying colonic polyps is the recommendation to at-risk family members with the APC gene variant. This screening would start from the early ages of 10 to 12 years. Genetic testing of children and adolescents would determine those that will require clinical screening (8, 17, 24). However, in regard to screening of brain tumors, there are no guidelines and proves to be arduous (11).

We suggest that the detection of early CRC and signs of FAP, via colonoscopy and biopsy, should highlight the need for genetic testing on samples. Identifying genetic variants can serve as screening for primary brain tumors. With identification of a primary brain tumor, detecting genetic variants may be imperative, allowing for screening and prevention of CRC. The discovery of a genetic variant associated with cancer predisposition syndromes will allow patients with an increased risk, and their families, to be monitored for developing manifestations (11).

Overall, the diagnosis and management of our index case illustrates a few key learning points. Firstly, it highlights the importance of taking a thorough history, particularly focusing on family history to identify potential cancer predisposition syndromes. It also highlights the need for expertise in linking a family history, a cancer predisposition syndrome and navigating testing to validate these complex diagnoses. In our index case, identifying two generations of colonic disease in the setting of medulloblastoma helped identify a potential germline FAP which prompted genetic testing. This case illustrates the presence of complex disorders that exist in low-and-middle income countries and the need for highly specialized testing such as molecular and genetic studies to facilitate more accurate diagnoses. That being said, the number of these rare cases will be substantially lower and discussions of incorporating such highly specialized testing will have to be further assessed based on financial feasibility of institutions. However, collaborative initiatives such as SCI are crucial in facilitating highly specialized testing to be done in high-income countries to help diagnose these very rare cases and aid in precision medicine.

## 4 Conclusion

Although TS2 has been documented in literature, albeit a rare syndrome, this case highlights a few essential points in ensuring the holistic care of children with cancer. It must be appreciated that some childhood cancers are due to cancer predisposition syndromes which are familial. As such, a detailed family history may be the only information needed to identify a predisposition syndrome, which is essential to all clinicians.

## Data availability statement

The original contributions presented in the study are included in the article, further inquiries can be directed to the corresponding author/s.

## Ethics statement

Ethical approval was not required for the study involving human samples in accordance with the local legislation and institutional requirements because no human samples were utilized in this study. Written informed consent for participation in this study was provided by the participants’ parents/legal guardians. Written informed consent was obtained from the individual(s) for the publication of any potentially identifiable images or data included in this article.

## Author contributions

MD-A: Writing – review & editing, Writing – original draft, Visualization, Conceptualization. BG: Writing – review & editing, Writing – original draft, Conceptualization. UB: Writing – review & editing, Investigation. CB: Writing – review & editing, Investigation. KD: Writing – review & editing, Writing – original draft, Supervision, Investigation, Conceptualization.

## References

[1] Zahnreich S Schmidberger H . Childhood cancer: occurrence, treatment and risk of second primary Malignancies. Cancers (Basel). (2021) 13:2607. doi: 10.3390/cancers13112607 34073340 PMC8198981

[2] Reece-Mills M Bodkyn C Baxter J-AB Allen U Alexis C Browne-Farmer C . Developing a partnership to improve health care delivery to children <18 years with cancer and blood disorders in the English-speaking Caribbean: lessons from the SickKids-Caribbean Initiative (SCI). Lancet Reg Health Am. (2023) 26:100592. doi: 10.1016/j.lana.2023.100592 37727865 PMC10506063

[3] Subramanian S Ahmad T . Childhood brain tumors, in: StatPearls (2023). Treasure Island (FL: StatPearls Publishing. Available online at: http://www.ncbi.nlm.nih.gov/books/NBK535415/ (Accessed October 29, 2023).

[4] Muskens IS Zhang C de Smith AJ Biegel JA Walsh KM Wiemels JL . Germline genetic landscape of pediatric central nervous system tumors. Neuro Oncol. (2019) 21:1376–88. doi: 10.1093/neuonc/noz108 PMC6827836 31247102

[5] Turcot J Despres JP St Pierre F . Malignant tumors of the central nervous system associated with familial polyposis of the colon: report of two cases. Dis Colon Rectum. (1959) 2:465–8. doi: 10.1007/BF02616938 13839882

[6] Walker DA Aquilina K Spoudeas H Pilotto C Gan H-W Meijer L . A new era for optic pathway glioma: A developmental brain tumor with life-long health consequences. Front Pediatr. (2023) 11:1038937. doi: 10.3389/fped.2023.1038937 37033188 PMC10080591

[7] Sloan EA Hilz S Gupta R Cadwell C Ramani B Hofmann J . Gliomas arising in the setting of Li-Fraumeni syndrome stratify into two molecular subgroups with divergent clinicopathologic features. Acta Neuropathol. (2020) 139:953–7. doi: 10.1007/s00401-020-02144-8 PMC7183424 32157385

[8] Taylor MD Northcott PA Korshunov A Remke M Cho Y-J Clifford SC . Molecular subgroups of medulloblastoma: the current consensus. Acta Neuropathol. (2012) 123:465–72. doi: 10.1007/s00401-011-0922-z PMC3306779 22134537

[9] Nemes K Bens S Bourdeaut F Johann P Kordes U Siebert R . Rhabdoid tumor predisposition syndrome, in: GeneReviews® (1993). Seattle (WA: University of Washington, Seattle. Available online at: http://www.ncbi.nlm.nih.gov/books/NBK469816/ (Accessed August 27, 2023). 29215836

[10] Pfister S Janzarik WG Remke M Ernst A Werft W Becker N . BRAF gene duplication constitutes a mechanism of MAPK pathway activation in low-grade astrocytomas. J Clin Invest. (2008) 118:1739–49. doi: 10.1172/JCI33656 PMC2289793 18398503

[11] Cancer Genome Atlas Research Network . Comprehensive genomic characterization defines human glioblastoma genes and core pathways. Nature. (2008) 455:1061–8. doi: 10.1038/nature07385 PMC2671642 18772890

[12] Carta R Del Baldo G Miele E Po A Besharat ZM Nazio F . Cancer predisposition syndromes and medulloblastoma in the molecular era. Front Oncol. (2020) 10:566822. doi: 10.3389/fonc.2020.566822 33194646 PMC7658916

[13] Khattab A Monga DK . Turcot syndrome, in: StatPearls (2023). Treasure Island (FL: StatPearls Publishing. Available online at: http://www.ncbi.nlm.nih.gov/books/NBK534782/ (Accessed October 29, 2023). 30521203

[14] Ullah F Pillai AB Omar N Dima D Harichand S . Early-onset colorectal cancer: current insights. Cancers (Basel). (2023) 15:3202. doi: 10.3390/cancers15123202 37370811 PMC10296149

[15] Hirsch S Dikow N Pfister SM Pajtler KW . Cancer predisposition in pediatric neuro-oncology-practical approaches and ethical considerations. Neurooncol Pract. (2021) 8:526–38. doi: 10.1093/nop/npab031 PMC8475219 34594567

[16] Dipro S Al-Otaibi F Alzahrani A Ulhaq A Al Shail E . Turcot syndrome: a synchronous clinical presentation of glioblastoma multiforme and adenocarcinoma of the colon. Case Rep Oncol Med. (2012) 2012:720273. doi: 10.1155/2012/720273 23119205 PMC3479943

[17] Ozerov SS Zakharov IV Talypov SR Konovalov DM Kisliakov AN Kachanov DI . Turcot syndrome. Rare observation and literature review. Zh Vopr Neirokhir Im N N Burdenko. (2013) 77:49–53; discussion 53. 23866578

[18] Skomorowski M Taxier M Wise W . Turcot syndrome type 2: medulloblastoma with multiple colorectal adenomas. Clin Gastroenterol Hepatol. (2012) 10:A24. doi: 10.1016/j.cgh.2012.06.013 22732270

[19] Hamilton SR Liu B Parsons RE Papadopoulos N Jen J Powell SM . The molecular basis of Turcot’s syndrome. N Engl J Med. (1995) 332:839–47. doi: 10.1056/NEJM199503303321302 7661930

[20] Mahapatra S Amsbaugh MJ . Medulloblastoma, in: StatPearls (2023). Treasure Island (FL: StatPearls Publishing. Available online at: http://www.ncbi.nlm.nih.gov/books/NBK431069/ (Accessed August 27, 2023).

[21] Yokota N Nishizawa S Ohta S Date H Sugimura H Namba H . Role of Wnt pathway in medulloblastoma oncogenesis. Int J Cancer. (2002) 101:198–201. doi: 10.1002/ijc.10559 12209999

[22] Amayiri N Swaidan M Ibrahimi A Hirmas N Musharbash A Bouffet E . Molecular subgroup is the strongest predictor of medulloblastoma outcome in a resource-limited country. JCO Glob Oncol. (2021) 7:1442–53. doi: 10.1200/GO.21.00127 PMC8492378 34609903

[23] Dahmen RP Koch A Denkhaus D Tonn JC Sörensen N Berthold F . Deletions of AXIN1, a component of the WNT/wingless pathway, in sporadic medulloblastomas. Cancer Res. (2001) 61:7039–43. 11585731

[24] Pakakasama S Tomlinson GE . Genetic predisposition and screening in pediatric cancer. Pediatr Clin North Am. (2002) 49:1393–413. doi: 10.1016/s0031-3955(02)00095-0 12580371

